# Effects of social media use on employment anxiety among Chinese youth: the roles of upward social comparison, online social support and self-esteem

**DOI:** 10.3389/fpsyg.2024.1398801

**Published:** 2024-08-16

**Authors:** Ting Jin, Yanshan Chen, Ke Zhang

**Affiliations:** School of Communication, Soochow University, Suzhou, China

**Keywords:** social media use, upward social comparison, online social support, self-esteem, employment anxiety

## Abstract

Based on the continuous development of new media and mobile communication technologies, social media has significantly influenced people’s daily thoughts and behaviors. For young people, social media is a platform for social interaction, and studies have found that social media helps Chinese graduates find employment. This study examined how social media use and its related factors affect employment anxiety. The data was collected from 1,204 Chinese youth through an online survey. The results showed that: (1) the intensity of social media use had a positive predictive effect on employment anxiety; (2) upward social comparison and online social support individually separately mediate the positive relationship between the intensity of social media use and employment anxiety; and (3) self-esteem negatively moderates the effect of upward social comparison and effect of online social support on employment anxiety. The study underscores the importance of reasonable social media usage strategies, positive social perception, and healthy self-perception for fostering a positive employment mindset among youth.

## 1 Introduction

Driven by new media and mobile communication technology, social media significantly influences people’s daily thoughts and behaviors. Global social media users increased by nearly 30%, adding over 1 billion in three years since COVID-19 started. Daily use rose by 2%, reaching 2 h and 31 min, peaking in 2022 (DataReportal, 2023). For Chinese youth, social media serves as a virtual space shaping their cognition and emotions, as well as a platform for social interaction, including employment. Social media use has been found to contribute to the employability outcomes of Chinese graduates (He et al., 2017).

However, China is confronted with a serious issue of youth unemployment. The estimated number of graduates from higher education institutions nationwide in 2023 is expected to reach 11.58 million, representing an increase of 820,000 compared to 2022 (Chinese Ministry of Education, 2022). At the same time, China’s youth unemployment rate has remained at a relatively high level, reaching 21.3% in June 2023, marking a new high since 2018 (National Bureau of Statistics of China, 2023). A change that may indicate worsening situations is that the Statistics Bureau announced the suspension of publishing youth unemployment rates (National Bureau of Statistics of China, 2023) after a strict investigation into the falsification of graduate employment data demanded (Chinese Ministry of Education, 2023). In such an employment environment filled with uncertainties and intense competition, it is inevitable for the youth to experience employment anxiety. A survey conducted among Chinese university students found that 40.46% of college students have a strong sense of job crisis, and 73.32% of them believe that the employment situation under COVID-19 is unpromising or average (Zheng et al., 2022). Employment anxiety has become a serious problem that urgently needs to be addressed.

In previous research, researchers have confirmed that social media is related to anxiety. Most studies have investigated the relationship between problematic social media use and anxiety (Lee-Won et al., 2015; Lopes et al., 2022), as well as specific types of anxiety such as social anxiety (Al-Ali et al., 2011; Stanculescu and Griffiths, 2022), and personal traits related to anxiety such as anxious attachment style (Blackwell et al., 2017; Stanculescu and Griffiths, 2022). Besides, while research has explored the factors influencing employment anxiety (Shin, 2019; Belle et al., 2022), few studies examined the association between social media use and employment anxiety, indicating a gap in this field that need to be filled. To investigate how social media use affects employment anxiety, this study introduces upward social comparison and online social support as mediating variables, and self-esteem as a moderating variable in the relationship between social media use and employment anxiety.

This paper examines the following questions: (1) How does social media use influence employment anxiety among Chinese youth? (2) What roles do related variables play in this effect? The results will help us understand the relationship between social media use and employment anxiety including the influencing mechanisms, and expand the empirical applications of social comparison theory and social support theory. The results can also provide suggestions for youth groups to reduce employment anxiety and enhance job outcomes.

## 2 Literature reviews and hypotheses development

### 2.1 Social media use and employment anxiety

As an online media based on Web 2.0 technologies, social media provides a virtual space for users to share content and build relationships (Mayfield, 2008; Kaplan and Haenlein, 2010; Peng, 2013). Users’ engagement with social media platform features is defined as social media use (SMU), and the intensity of social media use (ISMU) serves as the fundamental framework when researchers describe behavior patterns and emotional experiences of social media use, indicated by factors such as time spending on social media, frequency of usage, number of online friends, and online emotional bond strength (Hart et al., 2015).

Anxiety is a negative emotional state, a mixture of feelings such as fear, tension and worry, arising from nonspecific threats or vague perceptions of future risks (Smith and Lazarus, 1990; Lang et al., 2005). Employment anxiety (EA) is a specific type of anxiety, triggered by employment situations with uncertainty.

The research exploring the relationship between intensity of social media use and anxiety indicates that intensity of social media use can have effects on various types of anxiety as a trigger or elevator, directly or indirectly. A typical manifestation is the increase in anxiety caused by social media overload during the pandemic (Wang et al., 2023). Sufficient research can demonstrate the enhancing effect of social media use on FOMO (Fear of Missing Out) (Zhang et al., 2021). Simultaneously, the intensity of social media use can indirectly elevate anxiety through certain factors like risk perception, for example, women exposed to more social media content tend to be more sensitive in perceiving reproductive risks, leading to increased fertility-related anxiety (Liu and Song, 2022). Studies also suggest that daily social media use adversely impacts subjective well-being over time (Wirtz et al., 2021). Besides, a survey conducted during the COVID-19 period found that there is a significant negative correlation between social media usage and subjective happiness (Khodabakhsh and Ahmadi, 2020).

In sum, existing studies can confirm that the intensity of social media use can evoke negative emotions like fear of missing out and loneliness, as well as specific types of anxiety such as fertility-related anxiety and social anxiety, especially in uncertain environments. It can also be harmful to subjective well-being and happiness. Therefore, the first hypothesis is proposed as follows:

*H1:* The intensity of social media use positively affected Chinese youth’s employment anxiety.

### 2.2 The mediating role of upward social comparison

Upward social comparison (USC) is a concept within Social Comparison Theory. The theory posits that individuals, driven by the need for self-evaluation, tend to compare themselves to others in terms of opinions and abilities (Lemaine and Schachter, 1960). Upward social comparison is a type of social comparison where individuals compare themselves to others who are more advantaged than themselves (Suls and Wheeler, 2012). Upward social comparison can lead to two distinct effects: the assimilation effect causes individuals to psychologically align themselves with the comparator, while the contrast effect directs individuals to focus on the differences (Xing and Yu, 2006).

Social media fosters an environment conducive to upward social comparison due to its encouragement of positive self-presentation content (Seidman, 2013), leading researchers to closely associate upward social comparison with social media use. With the examined effect on positive emotions and the stimulating effect on negative emotions, upward social comparison is often identified as a mediating variable in studies exploring the impact of social media use on negative emotions. A study confirmed that with Upward social comparison and self-esteem as mediations, intensity of social media use shows a negative impact on subjective well-being (Wang et al., 2019). Furthermore, an investigation found that the intensity of social media use can not only increase anxiety through upward social comparison but also impact anxiety through sequential mediation involving upward social comparison and psychological capital (Qiu et al., 2017).

In conclusion, the examined studies highlight the mediating role of upward social comparison in the relationship between social media use and emotional outcomes, revealing its beneficial effects on negative emotions and specific types of anxiety. Therefore, the second hypothesis is proposed as follows:

*H2:* Upward social comparison mediates the relationship between intensity of social media use and Chinese youth’s employment anxiety, that is, intensity of social media use first enhances upward social comparison and then increases employment anxiety.

### 2.3 The mediating role of online social support

Online social support (OSS) is a concept within Social Support Theory. Social Support Theory posits that individuals can receive emotional and material support from social networks in communities, organizations, and society, helping them access resources and assistance to cope with challenges (Cullen, 1994). The main effect model from the theory suggests that social support systems bring positive emotional experiences to individuals (Wang, 2004), while the buffering effect model proposes that social support alleviates individuals’ perception of stress and negative emotional experiences under pressure (Kawachi and Berkman, 2001). Online social support is a form of social support, known as a sense of identity and belonging that individuals experience when understood and respected during emotional, informational, and material exchanges in virtual spaces (Liang and Wei, 2008).

Social media creates an online community for users, where they can also form social relationships and gain support. Online self-disclosure can positively impact the well-being of lonely individuals through online social support (Lee et al., 2013). For college students, the number of Facebook friends is associated with a stronger perception of social support, which in turn is correlated with reduced stress, decreased physical illness, and enhanced well-being (Nabi et al., 2013). Similarly, a survey of undergraduate students indicates a significant mediating role of social support and self-esteem in the relationship between active use of social networking sites and feelings of loneliness, including the direct mediating effect of social support and the sequential mediating effects of social support followed by self-esteem (Lin et al., 2022). Additionally, a study on the relationship between social support, occupational stress, and anxiety has demonstrated that social support can directly reduce anxiety (Beehr and McGrath, 1992).

In sum, existing studies suggest that the intensity of social media use can contribute to online social support, leading to enhanced happiness and reduced feelings of loneliness, stress, and anxiety. Therefore, the third hypothesis is proposed as follows:

*H3:* Online social support mediates the relationship between the intensity of social media use and Chinese youth’s employment anxiety, that is, the intensity of social media use first enhances upward social comparison and then reduces employment anxiety.

### 2.4 The moderating role of self-esteem

Self-esteem (SE) is the perception of an individual’s value, reflecting the difference between the individual’s perception of the self and the ideal self, representing the perception of one’s value (James, 1924; Rosenberg, 1962). As a personal trait, self-esteem often serves as a moderating variable in psychological or behavioral research.

Existing research on self-esteem has shown its buffering effects protecting individuals from emotional challenges, as well as its relationship with social comparison and social support. According to the anxiety buffer hypothesis, self-esteem hampers anxiety and depressive symptoms by acting as a shield against fear and loneliness (Greenberg et al., 1992). Individuals with low self-esteem experience higher levels of negative emotions when faced with negative social stimuli (Richter and Ridout, 2011). For young adults, self-esteem moderated the association between body-related guilt and the frequency of depressive symptoms, suggesting self-esteem is beneficial in alleviating depressive symptoms caused by body-related guilt (Brunet et al., 2019). Furthermore, self-esteem significantly moderated the impact of social comparison on body esteem among female college students: the low self-esteem group exhibited a contrast effect, while the high self-esteem group demonstrated an assimilation effect (Jones and Buckingham, 2005). Additionally, adolescents with higher self-esteem perceive social support better, thereby promoting their psychological well-being (Poudel et al., 2020). Among international students in China, compared with students with low self-esteem, the predictive effect of social support on attachment closeness was enhanced in those with high self-esteem (Li et al., 2021).

In sum, self-esteem acts like a shield, protecting individuals by buffering the impact of negative emotions. It also functions like an amplifier, magnifying the beneficial effects of social support. Additionally, self-esteem moderates the impact of social comparison. Therefore, the fourth hypothesis is proposed as follows:

*H4:* Self-esteem moderates the effect of upward social comparison on employment anxiety (H4a), and the effect of online social support on employment anxiety (H4b).

**Figure 1:**
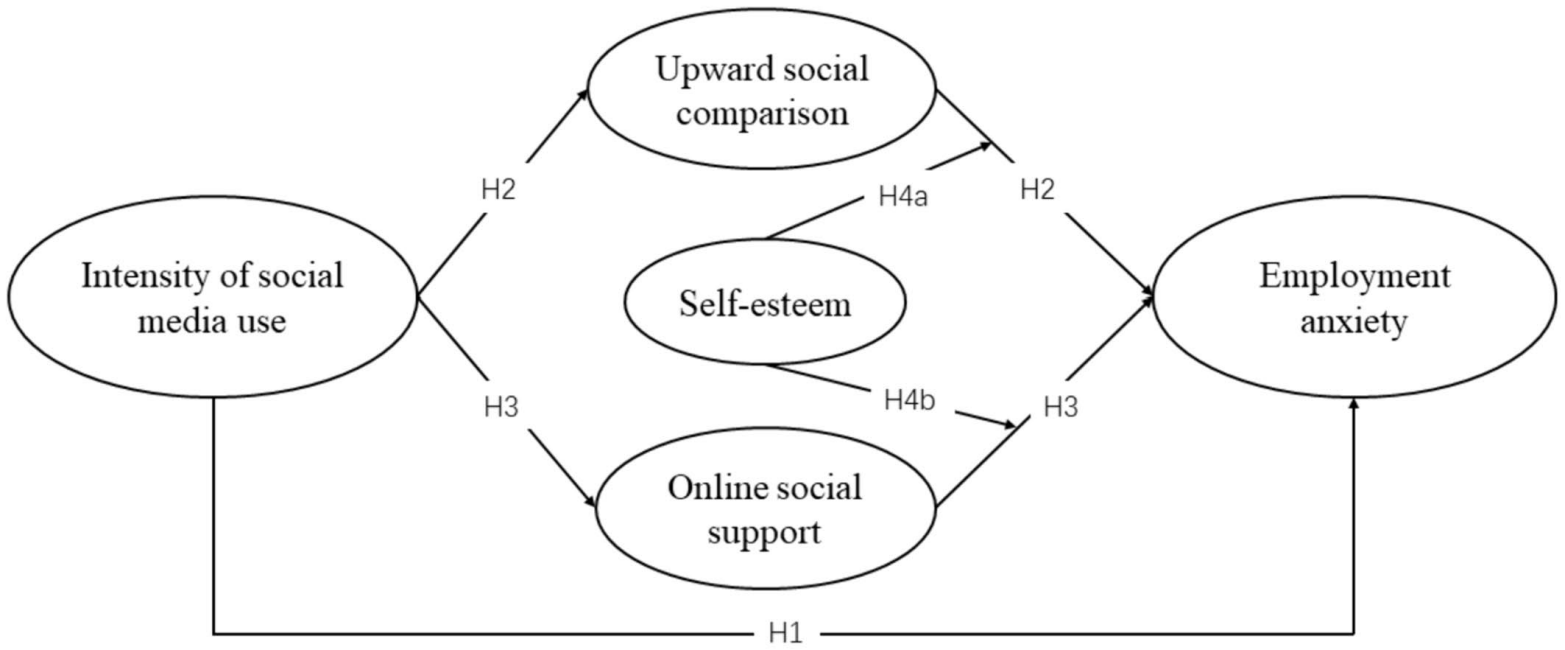
Conceptual model.

The conceptual model of this study is proposed in Figure 1.

## 3 Methods

### 3.1 Participants

This study was conducted through an online questionnaire based on Wenjuanxing (WJX), the largest Chinese online survey platform, in March 2023. A pre-test was conducted before the official test (*n* = 55, 48.2% male, 51.8% female).

We selected the overlapping range of youth definition (age 14–44) from the World Health Organization and working population definition (age 18–65) in China, defining youth in this study as 18–44 years old, with the number of about 511 million (National Bureau of Statistics of China, 2020). We use the minimum sample size formula to calculate the minimum sample, which is 1,068.

We collected 1,442 questionnaires from January to March 2023. After data cleaning, there were 1,204 effective samples with 48.6% male and 51.4% female. Sample sources include 31 provinces or municipalities in China. To avoid duplication, users with the same IP address or the same computer/mobile phone device can only access the questionnaire once. There was a disclaimer in the debriefing reminding the respondents that the study was a piece of academic research. Upon completing the questionnaire, each participant receives a reward of 2 yuan (approximately 0.3 USD).

### 3.2 Measure

The questionnaire consisted of 94 questions. We investigated demographic characteristics at first, including major, grade of school, graduation or expected graduation time, employment experience, and employment intention as demographic characteristics. The measurement instruments used in this study were derived from established scales, and all passed the reliability and supported testing in pre-tests (Cronbach’s α > 0.8, KMO > 0.7). The scales were all scored on a 5-point Likert scale, with 1 to 5 representing the sequential increase in personal attitudes.

The intensity of social media use scale was adapted from the 8-item Facebook Intensity of Use Scale, developed by Ellison et al. (2007). Considering that Facebook is not widely used among Chinese youth, we replaced “Facebook” in all items with “social media,” for example, “Social media has become part of my daily routine,” “I feel out of touch when I have not logged onto social media for a while.” We noted in the prompts that social media in this scale is defined as the sum of common social media platforms in China including Little Red Book, WeChat, QQ, Weibo, Twitter, and Facebook.

The upward social comparison scale directly adopted the 6-item upward social comparison subscale of the Iowa-Netherlands Comparison Orientation Measure (INCOM) developed by Gibbons and Buunk (1999). Example items include, “If I want to find out how well I have done something, I compare what I have done with how others have done,” “I often compare how I am doing socially (e.g., social skills, popularity) with other people.”

The online social support scale utilized the 11-item online social support subscale from the Online-Offline Support Scale compiled by Wang and Wang (2013). This subscale was adapted from the Social Support Scale developed by Leung (2003). Example items include, “Some members in the online community you can turn to for suggestions on how to deal with a personal problem,” “Some members in the online community you can get together with for relaxation.”

The self-esteem scale directly adopted the 10-item Self-Esteem Scale (SES), originally developed by Rosenberg (1962) and revised by Chinese scholars Yang and Wang (2007). Items 5, 8, 9, and 10 are reverse-scored. Example items include, “I consider myself a valuable person, at least good as others,” “I think I have many good qualities.”

The employment anxiety scale was adapted from the Career Choice Anxiety Questionnaire for College Graduates developed by Zhang and Chen (2006). Since the study samples participants aged 18–44 among Chinese youth, we replace “college students” with “youths” in item 4, “Whenever I see the news media report on the youth employment situation, I get anxious.” Other example items include, “A failed job interview makes me feel disheartened,” “I worry that my abilities may not meet the employer’s expectations.”

The study confirmed data reliability and validity through common method bias, reliability, and supported tests. Harman one-way test indicated acceptable common method bias (26.189%) (Tang and Wen, 2020). Reliability (Cronbach’s α > 0.7) and validity (KMO > 0.7, Bartlett’s sig < 0.01) tests supported scale consistency and applicability, reinforcing data credibility. The value of Cronbach’s α for each scale is shown in Table 1.

**Table 1:** Descriptive statistics and correlation test.

	ISMU	USC	OSS	SE	EA
ISMU	*0.78*				
USC	0.32 **	*0.84*			
OSS	0.70 **	0.40 **	*0.91*		
SE	0.21**	−0.07 *	0.07 *	*0.78*	
EA	0.25 **	0.49 **	0.29 **	−0.52**	*0.96*
Mean	3.39	3.66	3.58	3.31	3.36
SD	0.68	0.73	0.76	0.52	0.81

*N* = 1,204. Cronbach’s α value is given in italics along the diagonal. **p < 0.01; *p < 0.05.

ISMU, intensity of social media use; USC, Upward social comparison; OSS, online social support; SE, self-esteem; EA, employment anxiety.

## 4 Results

### 4.1 Descriptive statistics and correlation test

The SPSS statistics version 26.0 was used in the analysis. Before hypothesis testing, we conducted descriptive statistics and a test of correlation. The significant correlations among the variables are evident, and collinearity is acceptable, indicating that hypothesis testing can be conducted. The results are shown in Table 1.

### 4.2 The direct effect of social media use on employment anxiety (H1)

We assumed that the intensity of social media use positively affected Chinese youth’s employment anxiety (H1). To test H1, we used intensity of social media use as the independent variable and employment anxiety as the dependent variable to conduct a simple linear regression analysis. We included age, gender, major, education, graduation time, employment experience, and employment intention as control variables to exclude their interference with the relationship between the independent variable and the dependent variables. Control variables are included as dummy variables. The result confirmed that the **i**ntensity of social media use increased employment anxiety (*β* = 0.26, *p* < 0.01) after including control variables (Δ*R*^2^ = 0.07). The results supported H1.

**Figure 2:**
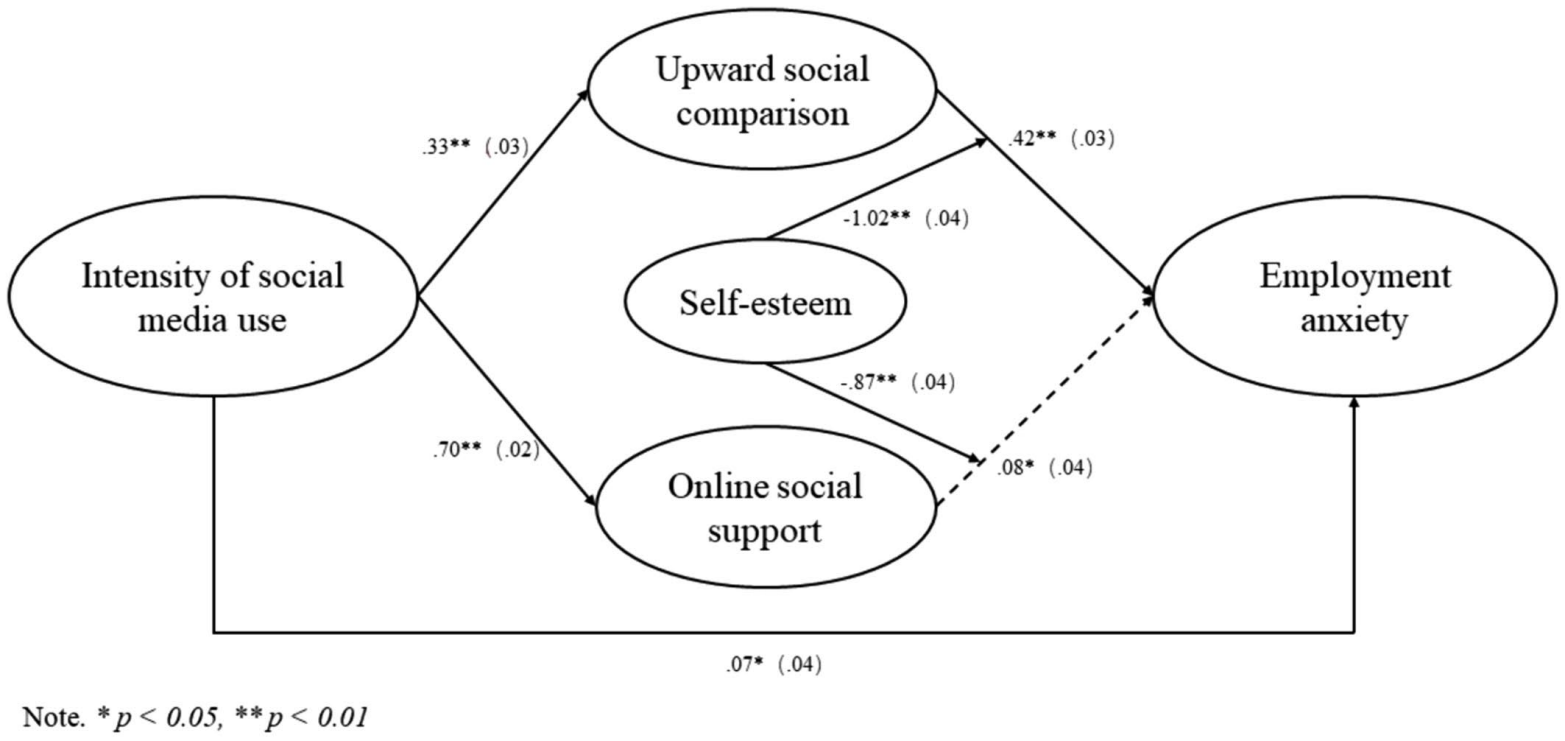
Statistical model.

Then we conducted path analysis to examine the relationships between variables. The model was specified with intensity of social media use as the predictor of employment anxiety, and included upward social comparison and online social support as mediators, as well as control variables such as age, gender, major, education, graduation time, employment experience, and employment intention, included as dummy variables as what we presented in Table 2. The path analysis results revealed that, the path from intensity of social media use to employment anxiety was significant (*β* = 0.07, SE = 0.04, *R*^2^ = 0.27, *p* = 0.04 < 0.05), the path from intensity of social media use to upward social comparison was significant (*β* = 0.33, SE = 0.03, *R*^2^ = 0.15, *p* = 0.00), the path from upward social comparison to employment anxiety was significant (*β* = 0.42,SE = 0.03, *R*^2^ = 0.27, *p* = 0.00), the path from intensity of social media use to online social support was significant (*β* = 0.70, SE = 0.02, *R*^2^ = 0.54, *p* = 0.00), and the path from online social support to employment anxiety was significant (*β* = 0.08, SE = 0.04, *R*^2^ = 0.27, *p* = 0.03). The results showed that each direct path among the independent variables, mediator variables and dependent variables was significant (*p* < 0.05), indicating that mediation tests can be further conducted. The path analysis results are shown in Figure 2.

**Table 2:** Regression analysis of the intensity of social media use on employment anxiety.

Model	Independent variable	B	*β*	*t*	*p*
1	Intensity of social media use	0.30	0.25	9.02	0.00
2	Intensity of social media use	0.32	0.26	9.41	0.00
	(Ctrl Var)				
	Major 1	0.04	0.02	0.59	0.55
	Major 2	0.20	0.09	2.76	0.01
	Major 3	−0.03	−0.01	−0.44	0.66
	Major 4	−0.13	−0.04	−1.29	0.20
	Major 5	0.00	0.00	0.03	0.97
	Education 1	0.05	0.03	0.94	0.35
	Education 2	0.25	0.08	2.72	0.01
	Education 3	−0.08	−0.04	−1.10	0.27
	Gender1	0.03	0.02	0.68	0.49
	Employment intention 1	0.06	0.03	1.08	0.28
	Employment intention 2	−0.17	−0.05	−1.76	0.08
	Graduation time 1	−0.06	−0.02	−0.68	0.50
	Graduation time 2	0.04	0.02	0.70	0.49
	Graduation time 3	0.06	0.03	0.82	0.41
	Graduation time 4	0.26	0.11	3.43	0.00
	Age 1	−0.07	−0.04	−1.18	0.24
	Age 2	−0.22	−0.08	−2.57	0.01
	Employment Experience 1	0.06	0.03	0.88	0.38
	Employment Experience 2	0.03	0.02	0.59	0.56
	Employment Experience 3	0.25	0.11	3.88	0.00
	Employment Experience 4	−0.13	−0.03	−1.11	0.27

Dependent variable: Employment anxiety; Control variables are included as dummy variables. The specific categories for the dummy variables are as follows:

Major: Reference category: Humanities majors; Major 1: Science majors; Major 2: Medical majors; Major 3: Engineering majors; Major 4: Art majors; Major 5: Others.

Education: Reference category: PhD; Education 1: Master; Education 2: Bachelor; − Education 3: Others.

Gender: Reference category: Male; Gender 1: Female.

Employment intention: Reference category: Intend to be employed now; Employment intention 1: Intend to be employed in the future; Employment intention 2: No employment intention or already employed.

Graduation time: Reference category: Before 2013; Graduation time 1: 2014–2019; Graduation time 2: 2020–2022; Graduation time 3: 2023 (Survey year); Graduation time 4: After 2023.

Age: Reference category: 18–24 years; Age 1: 25–34 years; Age 2: 35–44 years.

Employment Experience: Reference category: Current student with no experience; Employment Experience 1: Current student with internship experience; Employment Experience 2: Employed now; Employment Experience 3: Doing flexible work; Employment Experience 4: Unemployed now.

### 4.3 The mediation role of upward social comparison (H2)

We further assumed that the intensity of social media use would be indirectly associated with employment anxiety via upward social comparison (H2). The results of mediation analysis showed a total effect size of 0.30 (*p* < 0.05, 95%CI = [0.24, 0.37]), a direct effect of 0.13 (*p* < 0.05, 95%CI = [0.06, 0.19]), and a mediating effect of 0.18 (*p* < 0.05, 95%CI = [0.13, 0.22]), with the mediating effect accounting for 58.33% of the total effect. It can be seen that the intensity of social media use predicts employment anxiety via upward social comparison, that is the more frequent the use of social media, the stronger the upward social comparison young people perceive, and the higher their employment anxiety. Thus, H2 is supported. The results are shown in Table 3.

**Table 3:** Mediating effect of upward social comparison between intensity of social media use and employment anxiety.

Effect	Path	Effect size	95% CI		Effect ratio
			Lower	Upper	
Direct	Intensity of social media use – employment anxiety	0.13	0.06	0.19	41.67%
Mediation	Intensity of social media use – upward social comparison –employment anxiety	0.18	0.13	0.22	58.33%
Total		0.30	0.24	0.37	1

### 4.4 The mediation role of online social support (H3)

We also assumed that online social support mediates the relationship between the intensity of social media and employment anxiety (H3). The results of mediation analysis showed a total effect size of 0.30 (*p* < 0.05, 95%CI = [0.24, 0.37]), a direct effect of 0.11 (*p* < 0.01, 95%CI = [0.02, 0.20]), and a mediating effect of 0.20 (*p* < 0.05, 95%CI = [0.12, 0.27]), with the mediating effect accounting for 64.72% of the total effect. It can be seen that the intensity of social media use predicts employment anxiety via online social support, that is the more frequent the use of social media, the stronger the online social support young people perceive, and the higher their employment anxiety. Thus, H3 is partly supported. The results are shown in Table 4.

**Table 4:** Mediating effect of online social support between intensity of social media use and employment anxiety.

Effect	Path	Effect size	95% CI		Effect ratio
			Lower	Upper	
Direct	Intensity of social media use – employment anxiety	0.11	0.02	0.20	35.31%
Mediation	Intensity of social media use – upward social comparison –employment anxiety	0.20	0.12	0.27	64.72%
Total		0.30	0.24	0.37	1

### 4.5 The moderation roles of self-esteem (H4)

Considering the effects of individual psychological conditioning, we introduced self-esteem as a moderating variable into the model. We assumed that self-esteem moderates the effect of upward social comparison on employment anxiety (H4a), and the effect of online social support on employment anxiety (H4b).

**Figure 3:**
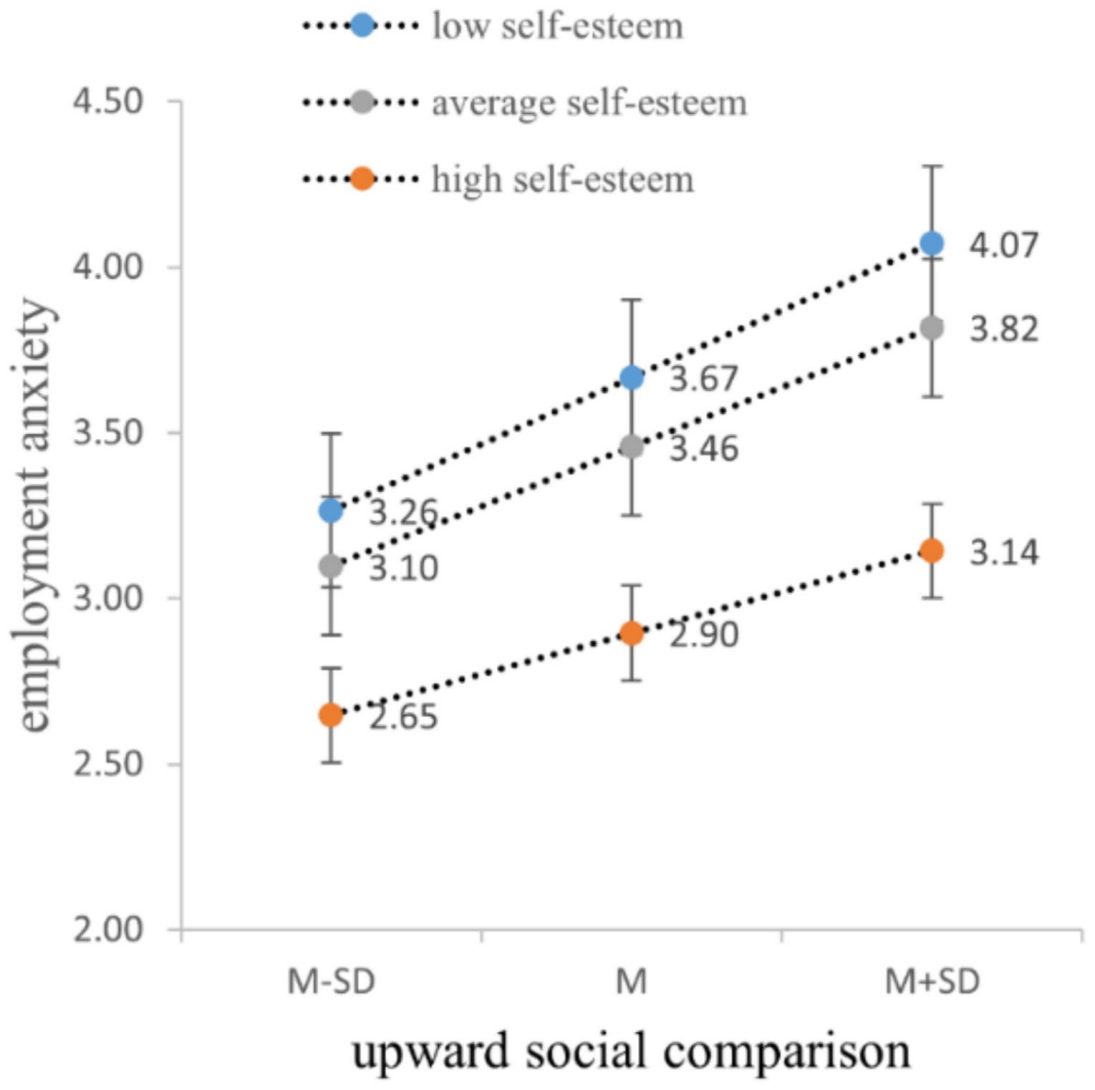
Moderating effect of self-esteem between upward social comparison and employment anxiety.

We first tested the moderating effect of self-esteem between upward social comparison and employment anxiety. The results indicate a significant increase in model fit (∆*R*^2^ = 0.01, *p* < 0.05) with the inclusion of the interaction term (upward social comparison × self-esteem). Self-esteem negatively moderates the effects of upward social comparison on employment anxiety (B = −0.23, *p* < 0.05). That is, compared to individuals with low self-esteem, the positive prediction of upward social comparison on employment anxiety of individuals with high self-esteem weakens. Thus, H4a is supported (Table 5). The slope graph is shown in Figure 3.

**Table 5:** Moderating effect of self-esteem between upward social comparison and employment anxiety.

Model	Variable	B	se	*β*	*t*	*p*	*R*^2^	△*R*^2^
Model 1	Const.	4.03	0.14		28.13	0.00	0.47	
	X	0.50	0.02	0.46	21.79	0.00		
	W	−0.76	0.03	−0.49	−23.13	0.01		
Model 2	Const.	1.21	0.51		2.38	0.02	0.49	0.01 (*p* < 0.05)
	X	1.28	0.14	1.16	9.43	0.00		
	W	0.08	0.15	0.05	0.56	0.58		
	XW	−0.23	0.04	−0.87	−5.81	0.00		

The independent variable X is upward social comparison, the moderation variable W is self-esteem, and the dependent variable is employment anxiety.

**Figure 4:**
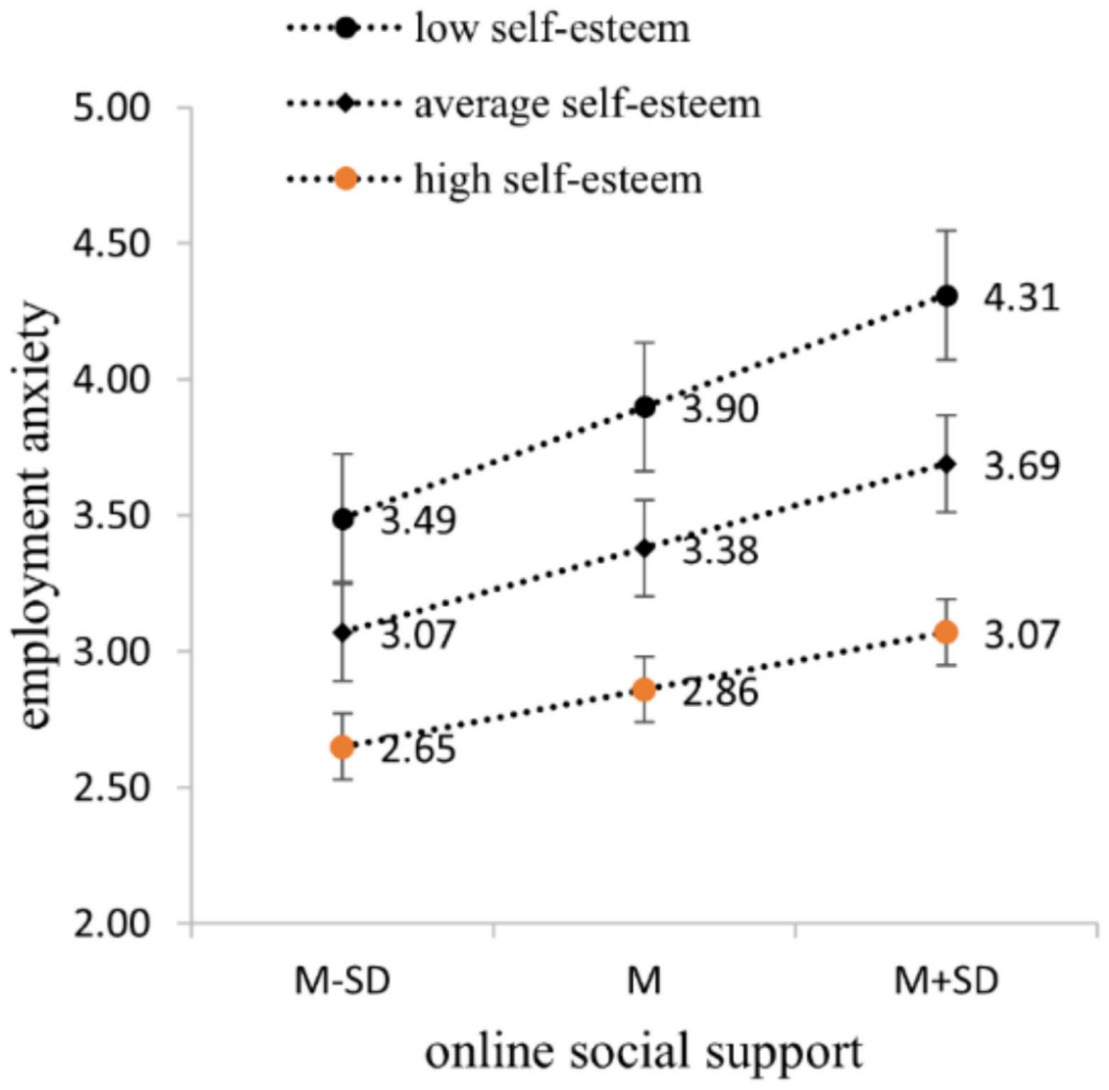
Moderating effect of self-esteem between online social support and employment anxiety.

Then we tested the moderating effect of self-esteem between online social support and employment anxiety. The results indicate a significant increase in model fit (∆*R*^2^ = 0.02, *p* < 0.05) with the inclusion of the interaction term (online social support × self-esteem). Self-esteem negatively moderates the effects of online social support on employment anxiety (B = −0.25, *p* < 0.05). That is, compared to individuals with low self-esteem, the positive prediction of online social support on employment anxiety of individuals with high self-esteem weakens. Thus, H4b is supported (Table 6). The slope graph is shown in Figure 4.

**Table 6:** Moderating effect of self-esteem between online social support and employment anxiety.

Model	Variable	B	se	*β*	*t*	*p*	*R*^2^	△*R*^2^
Model 1	Const.	4.9	0.13		37.54	0.00	0.43	
	X	0.44	0.02	0.41	18.62	0.00		
	W	−0.94	0.04	−0.60	−26.91	0.01		
Model 2	Const.	2.24	0.40		5.61	0.00	0.45	0.02 (*p* < 0.05)
	X	1.25	0.12	1.16	10.64	0.00		
	W	−0.10	0.13	0.06	−0.78	−0.34		
	XW	−0.25	0.04	−1.02	−7.02	0.00		

The independent variable X is online social support, the moderation variable W is self-esteem, and the dependent variable is employment anxiety.

In sum, H1, H2, and H4 are all supported, while H3 is partly supported. The statistical model is shown in Figure 2.

## 5 Discussion and conclusion

In this study, the regression test for H1 indicates that the intensity of social media use positively affects Chinese youth’s employment anxiety. The mediation test about upward social comparison for H2 shows that the intensity of social media use positively affects employment anxiety via upward social comparison, supporting H2. The mediation test about online social support for H3 reveals that the intensity of social media use positively affects employment anxiety via the mediation of online social support, contrary to the proposed negative mediation effect of online social support, and H3 is partly supported. The moderation test about self-esteem indicates that self-esteem weakens the significant effect of upward social comparison on employment anxiety, supporting H4a. It also shows that self-esteem weakens the significant effect of online social support on employment anxiety, supporting H4b.

These findings deepen our understanding of the relationship between social media use and employment anxiety as well as related variables. They can contribute to explaining the role of social media use in increasing negative emotions or specific types of anxiety (Zhang et al., 2021; Liu and Song, 2022; Wang et al., 2023), the bridging function of upward social comparison between social media use and anxiety or other negative emotions (Qiu et al., 2017; Wang et al., 2019), and the buffering effects of self-esteem when individuals encounter negative emotional stimuli (Greenberg et al., 1992; Richter and Ridout, 2011; Brunet et al., 2019). The results also provide an unexpected perspective on the negative role of online social support. The results of this study provide insights for Chinese youth on how to reduce employment anxiety by adjusting social media use strategies and mindsets. It also indicated what kind of characteristics are more susceptible to employment anxiety, that is youths with lower self-esteem find it more difficult to resist the increase in employment anxiety.

### 5.1 Impact of social media use on employment anxiety

One of the aims of this study was to discover the relationship between social media use and employment anxiety. We found that the intensity of social media use positively predicts employment anxiety both directly and indirectly through the mediating roles of upward social comparison and online social support, respectively. This is consistent with previous research focusing on the relationship between social media use and anxiety. For example, Wang et al. (2023) reported social media overload increased anxiety during COVID-19; Zhang et al. (2021) found that individuals with higher levels of social media use had higher levels of FOMO. Further, this study identified a significant relationship between social media use and employment anxiety among Chinese youth. This finding illustrates the importance of appropriate social media use strategies, such as reducing the frequency, duration, life integration, and psychological dependence on social media.

### 5.2 Upward social comparison in linking social media use with employment anxiety

Another purpose of this study was to discover the roles of related variables in the social media use-employment anxiety relationship. We found that both upward social comparison and online social support acted as mediating variables, acting as bridges to assist social media use in accomplishing the growth of employment anxiety. In this study, the mediating role of upward social comparison validates Social Comparison Theory: individuals’ perceived gap between themselves and their comparators in upward social comparison may cause envy and anxiety (Xing and Yu, 2006). It also aligns with prior research, for example, Qiu et al. (2017) reported that social media use increased college students’ anxiety through sequential mediation of upward social comparisons and psychological capital. This finding suggests that reducing upward social comparisons can reduce the employment anxiety experience associated with social media use.

### 5.3 Online social support in linking social media use with employment anxiety

However, the results of the mediating effect test of online social support were unexpected. Although the positive association between the extensity of social media use and online social support the Social Support Theory, which is that social media users receive online social support by engaging in virtual community interactions (White and Dorman, 2001), the positive association between online social support and employment anxiety was contrary to the findings of most researchers who reported the benefits of social support for individuals with negative emotions (Kawachi and Berkman, 2001; Beehr et al., 2010; Wang et al., 2019). Some studies suggest that in the context of social media, online social support may paradoxically contribute to increased levels of social media addiction, explaining the malfunction of social support. A survey among college students in Taiwan found a positive correlation between online social support and Facebook addiction (Tang et al., 2016). A study exploring the relationship between online and offline social support and internet addiction found that online social support is positively correlated with internet addiction, while offline social support is negatively correlated with internet addiction (Wang and Wang, 2013). Furthermore, not all forms of supportive social interactions have positive effects. A study found that interactions draw individuals’ attention to workplace stress, assistance that makes the recipient feel inadequate or incompetent, and unwanted help may be associated with deterioration in both psychological and physical health (Beehr et al., 2010). This may explain the failure of the anxiety-relieving effects of online social support in this study. The findings suggest that youth should be aware of the “dark side” of online social support, and be wary of the potential risk of addiction or stress while seeking online employment support.

### 5.4 Self-esteem in adjusting the relationships among upward social comparison, online social support and employment anxiety

In addition, we confirmed the negative moderating role of self-esteem in the positive effects of upward social comparison and online social support on employment anxiety, respectively, validating the buffering role of self-esteem according to the anxiety-buffer hypothesis (Greenberg et al., 1992), and also in line with plenty research findings (Leary and Baumeister, 2000; Jones and Buckingham, 2005; Richter and Ridout, 2011; Brunet et al., 2019). For example, Richter and Ridout (2011) reported individuals with low self-esteem experience higher levels of negative emotions when faced with negative social stimuli. The finding indicates that people with low levels of self-esteem are more likely to struggle with employment anxiety.

Based on the continuous evolution of new media and mobile technologies, this study contributes to understanding the nuanced impact of social media on youth employment anxiety in China. The findings highlight several theoretical contributions: firstly, demonstrating that increased social media use intensifies employment anxiety underscores the need for balanced digital engagement strategies. Secondly, identifying upward social comparison and online social support as mediators elucidates how social media influences perceptions of employment prospects. Thirdly, by revealing self-esteem as a moderator, the study underscores its role in mitigating the negative effects of social media use on anxiety levels. These insights underscore the significance of promoting healthy digital behaviors and positive self-perception to cultivate a resilient employment mindset among young adults in contemporary society.

This study, while revealing insightful findings on the impact of social media on employment anxiety among Chinese youth, has several limitations worth noting. Firstly, the cross-sectional nature of the data limits causal inferences, as it is challenging to determine if increased social media use causes heightened employment anxiety or if anxious individuals are more prone to heavy social media engagement. Secondly, the reliance on self-reported survey data may introduce response bias, where individuals might over- or under-report their social media use or anxiety levels. Thirdly, the study focuses on a specific demographic (Chinese youth) and may not fully generalize to other cultural contexts, where the influence of social media on employment anxiety might differ. Lastly, the study explores a limited set of factors related to social media use; future research could investigate additional variables, such as social media content type, privacy concerns, and the role of influencers, in shaping employment anxiety.

## Data Availability

The raw data supporting the conclusions of this article will be made available by the authors, without undue reservation.
